# Postoperative bisphosphonate do not significantly alter the fusion rate after lumbar spinal fusion: a meta-analysis

**DOI:** 10.1186/s13018-021-02444-z

**Published:** 2021-04-29

**Authors:** Jun Mei, Xiaoxu Song, Xiaoming Guan, Dou Wu, Junjie Wang, Qiang Liu

**Affiliations:** 1 Shanxi Medical University, Taiyuan, China; 2 Shanxi Bethune Hospital, Shanxi Academy of Medical Science, No. 99, Longcheng Street, Taiyuan, 030032 Shanxi Province China; 3 Qingdao University, Qingdao, China

**Keywords:** Bisphosphonates, Spinal fusion surgery, Fusion rate, Osteoporosis, Meta-analysis

## Abstract

**Background:**

To evaluate the effect of postoperative BP treatment on improving the fusion rate after lumbar spinal fusion surgery by performing a meta-analysis of randomized controlled trials (RCTs) and other comparative cohort studies.

**Methods:**

A comprehensive search of PubMed, EMBASE, the Web of Science, and the Cochrane Central Register of Controlled Trials was performed for RCTs and other comparative cohort studies on the effect of BP treatment on improving the fusion rate after lumbar spinal fusion surgery. The primary outcome measures were the number of patients with bone formation grades A, B, and C at 12 months of follow-up; fusion rates at 12 and 24 months of follow-up; vertebral compression fracture (VCF) at 12 and 24 months of follow-up; pedicle screw loosening at 24 months of follow-up; and cage subsidence, the Oswestry disability index (ODI), and the visual analogue score (VAS) at 12 months of follow-up. The final search was performed in July 2020.

**Results:**

Seven studies with 401 patients were included. Compared with the placebo, BP treatment did not significantly alter the number of patients with bone formation grades A, B, and C, or the VAS at the 12-month follow-up or the fusion rates at the 12- and 24-month follow-ups. In addition, compared with the placebo, BPs significantly reduced the risks of VCF at the 12- and 24-month follow-ups, pedicle screw loosening at the 24-month follow-up, and cage subsidence and the ODI at the 12-month follow-up.

**Conclusions:**

Postoperative BPs do not clearly improve bone formation and the fusion rate, but they reduce VCF, cage subsidence, and loosening of pedicle screws after lumbar fusion surgery compared with the control treatment.

## Introduction

Due to the ageing population worldwide, the incidence of numerous diseases associated with ageing has increased; for instance, lumbar degenerative disease is a common condition in humans, and approximately 37% of the adult population is estimated to suffer from this pathological condition [1]. Surgery is usually an inevitable intervention for individuals who do not receive a sufficient benefit from nonsurgical management [2]. Spinal fusion surgery is the most common therapeutic approach for various lumbar degenerative diseases since this treatment relieves pain and resolves neurological symptoms [3, 4]. Successful spinal fusion requires bone formation and remodelling, which requires cooperation between osteoblasts and osteoclasts [5]. According to a classic study, bone formation is graded by CT scans: grade A denotes bridging bone binding to two vertebral bodies nearby, grade B denotes bridging bone binding to one of vertebral bodies nearby, and grade C denotes incomplete bridging. The bone formation grade is tightly associated with the fusion rate [6]. Fusion is defined as a bridging bone between the vertebral bodies either inside or outside of the cage. Solid fusion is defined as less than 5° of angular motion on flexion-extension radiographs at the fusion level combined with the presence of grade A or B bone formation on coronal multiplanar CT reconstruction scans [6]. However, with ageing, another disease, osteoporosis, also occurs in patients who receive spinal fusion surgery, and studies have suggested that osteoporosis markedly affects the process of bone formation and remodelling [7, 8]. Despite the development of instrumentation and techniques, nonunion after lumbar spinal fusion surgery remains a primary cause of spinal fusion failure, and managing osteoporosis appears to improve the fusion rate [9, 10], as verified by the administration of teriparatide [11].

Bisphosphonates (BPs) are stable pyrophosphate analogues that tightly bind to bone apatites [12]. BPs exert their effect by inhibiting osteoclast differentiation and activity, preventing bone resorption and reducing its turnover; these processes significantly reduce the risk of osteoporosis [13]. BPs are divided into three different generations. The first generation includes clodronate, etidronate, and tiludronate, because they do not contain nitrogen. The second generation includes alendronate and pamidronate, since they have amino-terminal groups. The third generation includes risedronate and zoledronate, as they contain a cyclic side chain [14]. In general, compared with the first generation, the second and third generations exhibit greater affinity for hydroxyapatite in bone and thus have advantages in improving bone metabolism [15]. Currently, most researchers and doctors suggest that BPs should be the first-line medication and advise patients with osteoporosis to receive BP treatment to control their decreased bone mass density (BMD) [16–18]. However, despite the high efficacy of BPs, their effects on managing osteoporosis and subsequently influencing the fusion rate after lumbar spinal fusion surgery remain controversial [19, 20]. Although several animal studies on spinal fusion have reported a positive effect of BPs [21, 22], two recent clinical studies concluded that preoperative BPs had no effect on the nonunion rates [20, 23], and one systematic review did not clearly determine whether BP therapy after surgery provided an added benefit after lumbar fusion surgery [24]. A recent meta-analysis has evaluated the effect of postoperative BPs on the fusion rate [25], but the major objective was comparing teriparatide and BPs, and the comparisons in this study were not sufficiently comprehensive.

We therefore evaluated the effect of postoperative BP treatment on improving the fusion rate after lumbar spinal fusion surgery by performing a comprehensive meta-analysis of the published data in randomized controlled trials (RCTs) and other comparative cohort studies.

## Materials and methods

### Search strategy

Two trained investigators systematically searched major online databases, including PubMed, EMBASE, the Web of Science, and the Cochrane Central Register of Controlled Trials, on July 13, 2020. The following terms were used while searching the databases and were arranged in different combinations: “interbody fusion”, “lumbar fusion”, “spinal fusion”, “bisphosphonate”, “alendronate”, “clodronate”, “etidronate”, “ibandronate”, “minodronate”, “neridronate”, “olpadronate”, “pamidronate”, “risedronate”, “tiludronic acid”, and “zoledronic acid”.

### Study identification and eligibility criteria

Two trained investigators independently screened the titles and abstracts in the electronic databases to identify possible eligible studies. Subsequently, the full text was read to include the final studies that met the following criteria: (1) candidates: patients who experienced any type of lumbar fusion surgery; (2) intervention: BP treatment with or without calcium and vitamin D supplements after surgery served as the experimental group, and placebo or standard treatment plus calcium and vitamin D supplements served as the control group; (3) outcome: desirable parameters that described surgical effects, including the fusion rate, vertebral compression fracture, bone formation grade, and other parameters; (4) type of studies: RCTs or comparative cohort studies; and (5) language of publication: English articles.

### Data extraction and assessment of risk of bias

The following data were extracted from the included studies by the two investigators mentioned above: name of the first author, publication year, study location, surgical methods, number of subjects allocated to each group, lumbar diseases and surgical levels in each study, number of male subjects in each group, mean age and body mass index (BMI) of each group, intervention methods used in each group, background treatment, preoperative lumbar spine BMD t-score in each group, preoperative Oswestry disability index (ODI) in each group, and follow-up duration. Any existing disagreement was resolved by discussion with a third investigator. The Cochrane risk of bias tool and the Newcastle-Ottawa Scale were used to assess the methodological quality of RCTs, and retrospective or prospective cohort studies, respectively [26, 27].

### Statistical analysis

The two investigators identified and recorded the following outcome parameters: number of patients with bone formation grades A, B, and C at 12 months of follow-up, fusion rates at 12 and 24 months of follow-up, vertebral compression fracture (VCF) at 12 and 24 months of follow-up, pedicle screw loosening at 24 months of follow-up, and cage subsidence, ODI, and visual analogue score (VAS) at 12 months of follow-up.

The statistical analysis was performed using RevMan 5.3 software. Odds ratios (OR) plus 95% confidence intervals (CIs) (calculated using a random effects model) and the mean differences (MD) plus 95% CIs (calculated using a fixed effects model) were calculated for dichotomous variables and continuous outcomes, respectively. *P* < 0.05 was deemed statistically significant. Chi-square (*χ*^2^) and *I*^2^ tests were used to identify heterogeneity, with *p*<0.05 and *I*^2^>50% considered indicators of heterogeneity. If heterogeneity in continuous outcomes existed, a random effects model was applied.

## Results

### Literature search

**Fig. 1:**
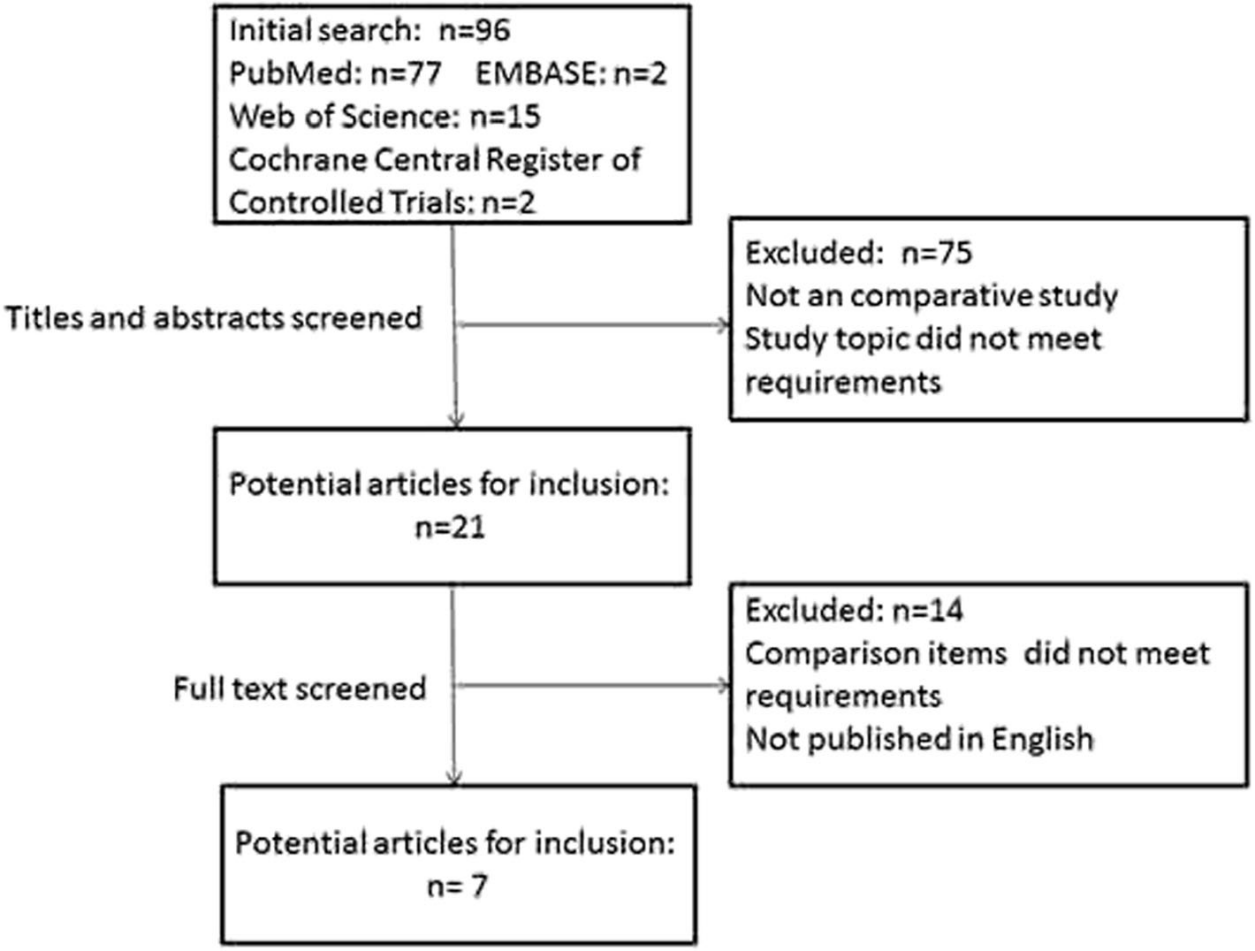
Flow diagram of included studies

Ninety-six titles were identified using our search terms, and duplicate articles were removed. Subsequently, 75 studies were eliminated after reading the titles and abstracts, leaving 21 trials for the full-text review. After reading the full text, 14 articles were excluded since their full text was not published in English or their comparison items did not meet the requirements. Finally, 7 articles were included in this meta-analysis [6, 19, 28–32] (Fig. 1).

### Study characteristics

Table 1 provides detailed information on the 7 included studies. The studies were published from 2011 to 2019. Four studies were RCTs [6, 19, 28–32], and 3 studies were retrospective comparative cohort studies [19, 29, 32]. The sample size ranged from 17 to 62 patients. Four hundred ninety-six patients with a mean age ranging from 63.63 to 77 years were included. One study did not mention the specific type of BPs used in their study. The shortest follow-up duration was 12 months, while the longest was 33.8 months.

**Table 1:** Characteristics of the 7 included studies

First author	Ken Nagahama	Chao Li	Seiji Ohtori	Sang-Mok Kim	Chao-Wei Tu	Fei Chen	Qirui Ding
Publication year	2011	2012	2013	2014	2014	2016	2017
Study location	Japan	China	Japan	South Korea	China	China	China
Surgical methods	Single-level PLIF	TLIF(with 23 one level, 16 two levels, 2 three levels in BPs group, 21 one level, 19 two levels, 1 three levels in control group)	Decompression and posterolateral fusion surgery at the level of spondylolisthesis	Single-level PLIF	Lumbar interbody fusion surgery (with 23 one level, 9 two levels in BPs group, 24 one level, 8 two levels in control group)	Single-level PLIF	TLIF
Number of patients in BPs/control group	19/17	41/41	20/22	22/22	32/32	33/36	30/34
Number of male patients in BPs/control group	1/1	13/16	0/0	9	5/6	6/7	3/4
Diseases type and number of diseases in BPs/control group	Degenerative spondylolisthesis (15), isthmic spondylolisthesis (1), foraminal stenosis (3)/degenerative spondylolisthesis (14), isthmic spondylolisthesis (1), foraminal stenosis (2)	Degenerative disc disease alone (26), isthmic or degenerative spondylolisthesis (12), recurrent disc herniations (3)/degenerative disc disease alone (25), isthmic or degenerative spondylolisthesis (14), recurrent disc herniations (2)	Lumbar degenerative spondylolisthesis with spinal stenosis	-	Degenerative lumbar spondylolisthesis	Single-level degenerative spondylolisthesis and diagnosis of osteoporosis	-
Number of operative levels in BPs/control group	L3-4 (1), L4-5 (14), L5-S1 (4)/L2-3 (1), L3-4 (3), L4-5 (12), L5-S1 (1)	L2-3 (1), L3-4 (7), L4-5 (27), L5-S1 (26)/L2-3 (1), L3-4 (5), L4-5 (31), L5-S1 (25)	-	-	-	L4-5 (24), L5-S1 (9)/L4-5 (25), L5-S1 (11)	-
Mean age of patients in BPs/control group	70.2/67.4	63.63 (SE 6.36)/63.83 (SE 5.70)	75 (SD 5)/77 (SD 5.8)	64.7 (range 60–74)	70.8 (SD 6.09)/69.7 (SD 6.02)	65 (SD 8)/63 (SD 7)	64.53 (SD 6.86)/66.44 (SD 6.44)
Mean BMI of patients in BPs/control group	-	23.01 (SE 3.53)/22.76 (SE 3.54)	-	-	31 (SD 2.1)/30 (SD 1.8)		23.98 (SD 2.32)/24.12 (SD 2.07)
Intervention methods in BPs/control group	Alendronate sodium 35 mg per week/ alfacalcidol 1 mg per day	An infusion of ZOL (5 mg, 100 ml) or physiological saline (100 ml) was administered 3 days after the surgery.	Risedronate2.5 mg per day for 10 months/no medication	Alendronate sodium 35 mg per week/no medication	Zoledronate 5 mg IV infusion 3 d after surgery and once-yearly thereafter/no medication	Zoledronic acid infusion (5mg), or the same volume of saline after surgery.	Intravenous zoledronic acid 5 mg at 3rd–5th days after operation/no medication
Background treatment	-	Calcium (1,000 mg/day) and vitamin D (400 IU/day) orally	-	-	-	Daily 1000 mg calcium and 800 IU vitamin D	Oral calcium 600 mg and vitamin D 800 IU
Preoperative lumbar spine BMD t-score in BPs/control group	−1.9/−2.2	Less than −1.5 (7), −1.5 to −2.5 (14), no less than −2.5 (20)/less than −1.5 (9), −1.5 to −2.5 (13),no less than −2.5 (19)	-	-3.75/-3.98	−3.1 (SD 0.59)/−2.9 (SD 0.5)	BMD of lumbar spine (L1-4) 0.709 (SD 0.003)g/cm^2^/0.698 (SD 0.004)g/cm^2^	-
Preoperative mean ODI scores in BPs/control group	20.3/21.6	-	36 (SD 10)/40 (SD 10)	-	63.5 (SD 6.3)/64 (SD 5.67)	20.8 (SD 2.6)/21.9 (SD 2.6)	39.2 (SD 2.27)/38.7 (SD 2.69)
Mean follow-up duration	12 months	12 months	1 year	33.8 months	24 months	1 year	30 months

*PLIF* posterior lumbar interbody fusion, *TLIF* transforaminal lumbar interbody fusion, *SD* standard deviation

### Study quality

**Fig. 2:**
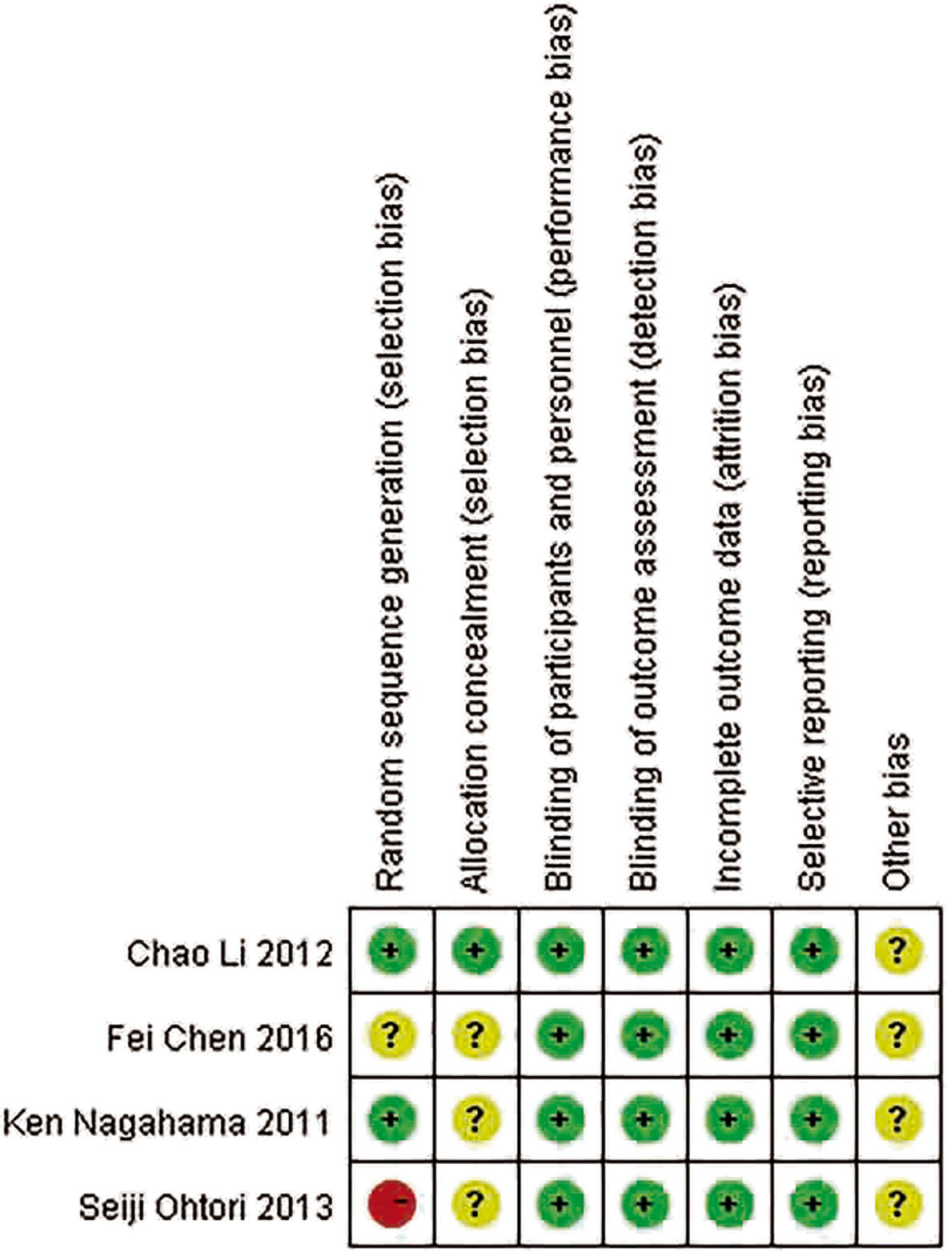
Risk of bias summary for included RCTs

The methodological quality of all included RCTs was high (Fig. 2), with a low risk of bias considered for most terms. All included cohort studies scored greater than 6 (Table 2), indicating a relatively high quality.

**Table 2:** Quality assessment of the included cohort trials

	Study (year)		
	Sang-Mok Kim (2014)	Chao-Wei Tu (2014)	Qirui Ding (2017)
**Selection**			
Representativeness of the exposed cohort	*	*	*
Ascertainment of exposure	*	*	*
Outcome not present at the start of the study	*	*	*
**Comparability**			
Comorbidities	*		
Other factors	*	*	*
**Outcome**			
Assessment of the outcome	*	*	*
Follow-up long enough for the outcome to occur	*	*	*
Adequacy of the follow-up	*	*	*
**Total**	8	7	7

According to Egger et al. [33], applying a funnel plot to assess publication bias is not credible for a meta-analysis that included fewer than 10 studies. Therefore, funnel plot was not used in this meta-analysis.

### Bone formation grade at the 12-month follow-up

The numbers of patients with bone formation grades A, B, and C were reported by more than one study. Table 3 shows the results of the forest plots, which indicated that BPs did not significantly influence bone formation grades A, B, and C at the 12-month follow-up compared with the control treatments. Random effect model was used to solve the heterogeneity.

**Table 3:** Results of the forest plots for the bone formation grade at the 12-month follow-up

Bone formation grade at the 12-month follow-up	Number of patients	Number of included studies	OR	95% CI	*P* value	*χ*^2^	*I*^2^	Effect model
Number of patients with bone formation grade A at 12 months of follow-up	105	2	1.32	0.61, 2.86	0.48	0.49	0%	Random effect
Number of patients with bone formation grade B at 12 months of follow-up	105	2	1.13	0.46, 2.75	0.79	0.33	0%	Random effect
Number of patients with bone formation grade C at 12 months of follow-up	105	2	0.41	0.04, 4.20	0.45	0.07	71%	Random effect

### Fusion rates at the 12- and 24-month follow-ups

Fusion rates at the 12- and 24-month follow-ups were provided reported by more than one study. Table 4 shows the results of the meta-analysis, which suggests that compared with the control treatments, BPs did not clearly alter influence the fusion rates at the 12- and 24-month follow-ups.

**Table 4:** Results of the forest plots for the fusion rates at the 12- and 24-month follow-ups

Fusion rate	Number of patients	Number of included studies	OR	95% CI	*P* value	*χ*^2^	*I*^2^	Effect model
Fusion rate at the 12-month follow-up	338	4	1.55	0.76, 3.17	0.23	0.32	14%	Random effect
Fusion rate at the 24-month follow-up	108	2	1.47	0.52, 4.13	0.47	0.21	36%	Random effect

### Number of patients with VCF at the 12- and 24-month follow-ups

The number of patients with VCF was reported by more than one study. Table 5 shows the results of the forest plots. Compared with the control treatments, BPs significantly reduced the risks of VCF at the 12- and 24-month follow-up visits.

**Table 5:** Results of the forest plots for the number of patients with VCF at the 12- and 24-month follow-ups

Number of patients with VCF	Number of patients	Number of included studies	OR	95% CI	*P* value	*χ*^2^	*I*^2^	Effect model
Number of patients with VCF at the 12-month follow-up	105	2	0.07	0.01, 0.59	0.01	0.96	0%	Random effect
Number of patients with VCF at 24-month follow-up	128	2	0.20	0.07, 0.58	0.003	0.54	0%	Random effect

### Number of patients with pedicle screw loosening at the 24-month follow-up

**Fig. 3:**
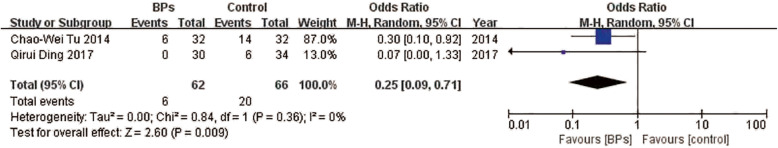
Forest plot showing the number of patients with pedicle screw loosening at the 24-month follow-up

The number of patients with pedicle screw loosening at the 24-month follow-up was reported by more than one study. As shown in Fig. 3, compared with control treatments, BPs significantly reduced the risks of pedicle screw loosening at the 24-month follow-up.

### Number of patients with cage subsidence

**Fig. 4:**
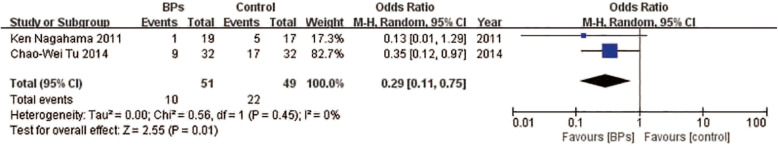
Forest plot showing the number of patients with cage subsidence

The number of patients with cage subsidence was described by more than one study. As shown in Fig. 4, compared with the control treatments, BPs significantly reduced the incidence of cage subsidence.

### ODI and VAS at the 12-month follow-up

The ODI and VAS at the 12-month follow-up were provided by more than one study. In Table 6, BPs did not noticeably alter the ODI and VAS compared with the control treatment. Random effect models were applied to solve the heterogeneity.

**Table 6:** Results of the forest plots for the ODI and VAS

Parameters	Number of patients	Number of included studies	MD	95% CI	*P* value	*χ*^2^	*I*^2^	Effect model
ODI at the 12-month follow-up	175	3	−1.98	−4.68, 0.72	0.15	0.10	56%	Random effect
VAS at 12-month follow-up	106	2	−0.34	−1.12, 0.44	0.39	0.05	74%	Random effect

### Subgroup analysis for RCTs and non-RCTS

Because the level of evidence is quite different between RCTs and non-RCTs, we separated the results of forest plots into RCTs and non-RCTs. Table 7 indicates that nearly all results were similar to those of the meta-analysis for both RCTs and non-RCTs, with the exception of the ODI score, which presents higher heterogeneity than that of the overall meta-analysis. A random effect model was used to solve the heterogeneity.

**Table 7:** Results of the forest plots for the subgroup analysis for RCTs and non-RCTs

Type of study	Comparative parameters	Number of patients	Number of included studies	OR	95% CI	*P* value	*χ*^2^	*I*^2^	Effect model
RCTs									
	Number of patients with bone formation grade A at 12 months of follow-up	105	2	1.32	0.61, 2.86	0.48	0.49	0%	Random effect
	Number of patients with bone formation grade B at 12 months of follow-up	105	2	1.13	0.46, 2.75	0.79	0.33	0%	Random effect
	Number of patients with bone formation grade C at 12 months of follow-up	105	2	0.41	0.04, 4.20	0.45	0.07	71%	Random effect
	Fusion rate at the 12-month follow-up	228	3	1.61	0.56, 4.67	0.38	0.18	42%	Random effect
	Number of patients with VCF at the 12-month follow-up	105	2	0.07	0.01, 0.59	0.01	0.96	0%	Random effect
	ODI at the 12-month follow-up	111	2	−1.61	−5.88, 2.67	0.46	0.03	78%	Random effect
Non-RCTs									
	Fusion rate at the 12-month follow-up	179	2	1.26	0.52, 3.05	0.62	0.46	0%	Random effect
	Fusion rate at the 24-month follow-up	108	2	1.47	0.52, 4.13	0.47	0.21	36%	Random effect
	Number of patients with VCF at 24-month follow-up	128	2	0.20	0.07, 0.58	0.003	0.54	0%	Random effect
	Number of patients with pedicle screw loosening at the 24-month follow-up	128	2	0.25	0.09, 0.71	0.009	0.36	0%	Random effect

### Sensitivity analysis

**Fig. 5:**
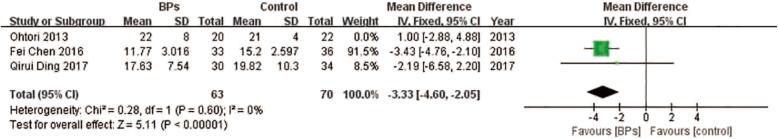
Results of the sensitivity analysis for the ODI at the 12-month follow-up. The study by Ohtori et al. was omitted, and the conclusions from the forest plot clearly changed. The forest plot shows that compared with the control treatment, BPs noticeably reduced the ODI at the 12-month follow-up

We conducted a sensitivity analysis to identify the source of heterogeneity in the comparison of the ODI between groups at the 12-month follow-up (Fig. 5). Due to the type of BPs, ratio of female patients, and age of patients, we omitted the study conducted by Ohtori et al., and the heterogeneity was clearly decreased and the result changed significantly. As shown in the forest plot, BPs clearly reduced the ODI at the 12-month follow-up compared with the control treatment.

## Discussion

Overall, the forest plots shown above suggest that compared with the placebo, BP treatment did not significantly alter the number of patients with bone formation grades A, B and C, or the VAS at the 12-month follow-up or the fusion rates at the 12- and 24-month follow-ups. In addition, compared with the placebo, BPs significantly reduced the risks of VCF at the 12- and 24-month follow-ups, pedicle screw loosening at the 24-month follow-up, and cage subsidence and the ODI at the 12-month follow-up.

Since the mechanism of BPs involves the inhibition of bone resorption, the BP treatment might modify the remodelling process associated with spinal fusion [34]. However, although a BP treatment increases bone formation after lumbar spinal fusion surgery in animal studies, the fusion rate is reduced [35–37]. In contrast, several clinical studies have documented positive results for the effect of BP treatment on bone formation and the fusion rate [6, 29], although a recent study observed a low fusion rate in patients with long-term BP treatment [20]. Our forest plots indicated no clear difference in bone formation and the fusion rate between patients treated with BPs and the control treatment. A study conducted by Nagahama et al. showed a positive effect of BP treatment on bone formation grade C and the fusion rate at the 12-month follow-up; the BP they used was alendronate. Another study that used alendronate was conducted by Kim et al., who did not observe a positive effect on the fusion rate at the 24-month follow-up; therefore, the type of drug may not have caused the positive result, and the follow-up time point may also have contributed [6, 19]. The reason for the negative results may be that although BPs inhibit bone resorption, they also in turn inhibit bone remodelling and may remain callus but may delay final remodelled-bone union. In addition, a study also suggested that BPs have potential antiangiogenic effects and reduce the blood supply at the fusion site [38]. The controversial conclusions regarding the effect of BPs on lumbar fusion among previous studies may result from different equivalent points of bone resorption and formation that BPs influenced in each study. Further studies are needed to confirm our hypothesis and results.

Osteoporosis-associated bone fragility (such as VCF and cage subsidence) and loosening of pedicle screws are the primary reasons for spinal fusion failure [8, 9, 39]. Although BP treatment did not significantly alter bone formation and the fusion rate, our forest plots suggested that the BP treatment significantly reduced the risks of VCF at the 12- and 24-month follow-ups and pedicle screw loosening at the 12-month follow-up. Therefore, BP treatment might exert a positive effect on these two complications. Two included studies provided detailed information about the number of patients with cage subsidence [6, 32]. Although the forest plot showed a positive result for BP treatment, the follow-up duration was not consistent between the two studies; therefore, the result may have also been influenced by the follow-up period. Nevertheless, in another study that compared alendronate and placebo, the researchers clearly observed a reduced length of cage subsidence in patients who received L4-5 lateral transpsoas interbody fusion [40]. However, due to the small sample size and use of different statistical methods, we were unable to conduct a meta-analysis on cage subsidence, and further studies are needed to explore these fields.

The ODI measures the degree of disability and estimates quality of life in a person with low back pain, while the VAS is a parameter that evaluates the degree of pain. According to our meta-analysis, compared with the placebo, BPs did not significantly alter the ODI and VAS at the 12-month follow-up, and thus they did not clearly improve quality of life. The ODI result is consistent with three other included studies, which also showed no difference between the two groups [6, 20, 30], and only one other included study indicated that BPs clearly reduced the ODI compared with control treatments [32]. Due to the lack of reporting of standard deviations in most studies, we were unable to conduct a meta-analysis that included these data. In addition, the heterogeneity of the two results was relatively high. In the sensitivity analysis, we omitted the study conducted by Ohtori et al. due to the type of BPs, female ratio and age instead of simply the study type, and the forest plot showed that BPs clearly reduced the ODI. Moreover, in the comparison of VAS at the 12-month follow-up, we were unable to conduct a sensitivity analysis and subgroup analysis due to the small sample size [29, 31], but the study conducted by Ding et al. showed a clear decrease in the VAS for patients treated with BPs, while the study conducted by Ohtori et al. showed a neutral result. The studies did not clearly determine whether the BP type, ratio of females, and age influenced the result. Further studies are needed to explore the effects of these parameters. Another clinical outcome that evaluated quality of life provided by included studies is Short Form 36 scores, which revealed a positive effect of BP treatment [29] and no clear difference [20], respectively. However, due to the small sample size, we were unable to perform a meta-analysis, and further studies are needed to explore the Short Form 36 scores. The mechanism of BPs includes an effect on osteoclasts. Two included studies described changes in two bone turnover markers, propeptides of type I collagen (PINP) and C-telopeptide of type I collagen (CTX), and their results indicated that BPs inhibit both bone formation and resorption [28, 30]. One included study also indicated that the BMD of the femoral neck was clearly increased in the BP treatment group [28]. However, we were unable to perform a meta-analysis due to differences in expression and small sample sizes, and further studies are required in this area.

Compared with a previous meta-analysis [25], we compared more parameters to evaluate the effect of BP therapy on lumbar fusion surgery and obtained some new findings, which indicated that compared with controls, BPs can significantly reduce VCF, cage subsidence, and loosening of pedicle screws after lumbar fusion surgery. Although the quality of included studies was relatively high, but we should also take consider the limitations listed below. First, the BPs used in the included studies were either alendronate or zoledronic acid, and other BP treatments had no effect on the fusion rate after lumbar spinal fusion surgery. Second, the main limitation of this meta-analysis is the small number of included studies and RCTs in particular, which may decrease the strength of our forest plots. Additionally, as the number of included studies was less than 10, meta-regression analyses and funnel plots were unable to be performed. Moreover, due to the small sample size of patients from RCTs, the data from the retrospective cohort studies may have influenced our results. Third, the included patients were mostly from East Asia, which may limit the wide application of this study. Finally, only English articles were considered eligible, which may have resulted in selection bias.

## Conclusions

Our meta-analysis of RCTs revealed that postoperative BPs do not clearly improve bone formation and the fusion rate, but they reduce VCF, cage subsidence, and loosening of pedicle screws after lumbar fusion surgery compared with the control treatment. In addition, the effect of BPs on the ODI and VAS remains inconclusive. Finally, the number of studies and RCTs included in our meta-analysis is small, and further RCTs involving larger sample sizes are required to confirm our results and provide additional evidence in this field.

## Data Availability

As a meta-analysis, there are no patient data sets.

## Abbreviations

BPs: Bisphosphonates
BMD: Bone mass density
RCTs: Randomized controlled trials
BMI: Body mass index
ODI: Oswestry disability index
VCF: Vertebral compression fracture
VAS: Visual analogue score
OR: Odds ratios
CIs: Confidence intervals
MD: Mean differences
PLIF: Posterior lumbar interbody fusion
TLIF: Transforaminal lumbar interbody fusion
SD: Standard deviation
